# Knowledge, attitudes and management skills of medical practitioners regarding weight management

**DOI:** 10.4102/phcfm.v8i1.1187

**Published:** 2016-11-29

**Authors:** Vangile B. Mkhatshwa, Gboyega A. Ogunbanjo, Langalibalele H. Mabuza

**Affiliations:** 1 Department of Family Medicine and Primary Health Care, Sefako Makgatho Health Sciences University, South Africa

## Abstract

**Background:**

Overweight and obesity have become a global problem. Health professionals are poorly prepared in weight management, which has an effect on their attitudes and management skills with regard to overweight and obese patients.

**Aim and setting:**

To assess the knowledge, attitudes and management skills of medical practitioners regarding weight management at Odi District Hospital, Gauteng Province, South Africa.

**Methods:**

We conducted a cross-sectional study on 48 medical practitioners at Odi Hospital between 01 October and 31 October 2013. A self-administered questionnaire was used to assess their knowledge, attitudes and management skills in weight management. The SPSS^®^ statistical software (Version 22) was used for data analysis. A *p* < 0.05 was considered significant.

**Results:**

Fifty medical practitioners were recruited, 48 consented to participate and 28 (58.3%) were male. Their categories were community service doctors (3), medical officers (21), registrars (22) and others (2). Thirty-seven (77.1%) never received training in weight management (*p* < 0.001). Thirty-two (66.7%) regarded weight management as not confined to a dietician (*p* < 0.001) and 27 (56.2%) regarded weight management as usually unsuccessful (*p* = 0.004). Forty-seven (97.9%) provided lifestyle modifications and 43 (89.6%) involved the patient’s family in weight management (*p* < 0.001). More non-registrars [14 (77.8%)] than registrars [8 (38.1%)] measured the body mass index (BMI) routinely (*p* = 0.013).

**Conclusion:**

Few medical practitioners received training in weight management. They regarded weight management as usually unsuccessful and lacked confidence in the same owing to lack of training. They provided lifestyle modifications and involved the patient’s family in weight management. Non-registrars measured the BMI routinely. There is a need for training in weight management at undergraduate and post-graduate levels.

## Background

Overweight and obesity have become a global problem.^1,2^ In 2010, it was estimated that overweight and obesity caused 3.4 million deaths and a loss of 3.9% of years of life globally.^3^ It has been suggested that unabated overweight and obesity could lead to a global decline in life expectancy in the United States^4^ as a result of the associated risk factors and comorbidities, such as cardiovascular disease (CVD), diabetes (type 2) and various cancers.^5^ The concern about health risks associated with obesity has led member states of the World Health Organization (WHO) to adopt a voluntary target to halt the rise in obesity by 2025.^6^

In keeping with the global trend, in recent years there has also been a rise in the prevalence of overweight and obesity in the African continent.^7,8^ South Africa has been reported to have the highest prevalence of overweight and obesity in Sub-Saharan Africa (SSA),^9^ which has been ascribed to socio-cultural, socio-economic and behavioural factors.^10^ In the late eighties, obesity was defined as ‘a condition of abnormal or excessive fat accumulation in adipose tissue, to the extent that health may be impaired’.^11^

Parameters commonly used to measure overweight and obesity are the body mass index (BMI), waist circumference (WC) and waist–hip ratio (WHR).^12^ The BMI (defined as the individual’s weight in kilograms, divided by the square of the height in metres (kg/m^2^)^11^ provides the most useful population-level measure of overweight and obesity, but it is a crude index as it does not account for the wide variation in body fat distribution.^13^ The BMI is classified as follows: underweight (<18.5 kg/m^2^), normal range (18.5–24.9 kg/m^2^), overweight (25.0–29.9 kg/m^2^), obese class I (30.0–34.9 kg/m^2^), obese class II (35.0–39.9 kg/m^2^) and obese class III (≥40.0 kg/m^2^).^11^

WC is a highly sensitive and specific measure of abdominal fat distribution and thus it is valuable for identifying overweight and obesity among patients at risk of developing metabolic complications. It is unrelated to height and correlates closely with BMI and WHR.^11^ The cut-off points are WC > 102 cm for men and >88 cm for women.^14^ WC has been proposed to be the best indicator in the measurement of central obesity, with excellent correlation with abdominal imaging and high association with CVD risk factors, especially diabetes.^15,16^ The WHR is also a measure of central obesity, since as the abdominal fat increases, so does the WC – relative to the hip girth.^17^ The cut-off points for defining central obesity are WHR > 1.0 in men and > 0.85 in women.^11,13^

The graded classification of overweight and obesity is valuable as it allows meaningful comparisons of weight status within and between populations, identification of individuals and groups at increased risk of morbidity and mortality as well as the identification of priorities for intervention at individual and community levels.^11^ In this study, we decided to use the BMI in the assessment of knowledge, attitudes and skills of medical practitioners as it is the most widely used measurement of overweight and obesity in all age groups.^16^

It has been shown that health professionals are poorly prepared to address overweight and obesity as a result of poor training in behavioural change strategies and the lack of experience in working within inter-professional teams.^18^ The central role of the primary health care practitioners (general practitioners and practice nurses) has been acknowledged in the United Kingdom and Scotland, resulting in the formulation of guidelines on overweight/obesity prevention and management at primary care level.^19^ The advantage of this strategy is the population coverage and continuity of care and trust built over a number of encounters,^20^ and the practical application of the primary care principle that every patient encounter should be regarded as an opportunity for health promotion.^21^ The Royal College of Physicians in the United Kingdom has issued a report on the training of general practitioners in weight management. The report has indicated that the training was minimal and poorly coordinated – ‘a reflection of lack of focus on obesity throughout medical training as a whole’.^22^

It has been shown that health care practitioners’ practice in the management of patients with overweight and obesity is influenced by their attitudes.^19^ In a study comparing weight bias in 2001 versus 2013 in the management of overweight and obesity among weight management specialists, it was demonstrated that there has been a paradigm shift from implicit (unconsciously expressed) to explicit (consciously expressed) anti-fat bias. The biases were expressed by the medical practitioners as bad, lazy, stupid and worthless.^23^ The weight of the health care practitioner may also influence their attitudes towards management of overweight and obesity as an overweight and obese practitioner would be less inclined to advise on weight management. In addition, an overweight and obese patient may not take seriously advice from a practitioner of similar weight.^19^

Literature on the training of health care practitioners in general, and medical practitioners in particular, in Africa is lacking. Most of the studies report on nutrition and management of patients with overweight and obesity.^24,25^ However, in South Africa, Malan, Mash and Everett-Murphy published work on development of a training programme to counsel patients on risky behaviour (including physical inactivity and unhealthy diet),^26^ and evaluation of a training programme for primary care providers on brief behavioural change counselling (BBCC) on risk factors for non-communicable diseases which could result from overweight and obesity.^27^

The aim of this study was to determine the knowledge, attitudes and management skills of medical practitioners in the management of overweight and obese patients at Odi District Hospital in the Gauteng Province. At the time of the study, the medical practitioners at the hospital comprised three categories: community service doctors, medical officers (MOs) and registrars (residents). In the South African health care system, a 2-year internship programme is followed by 1 year of community service, hence the title of community service doctor. The medical practitioner who has completed his/her community service year and is practicing in the public service is referred to as a MO. A registrar is a medical practitioner who is pursuing specialist training in a particular medical discipline; General Surgery, Family Medicine, and so on.^28^ In this study, all the registrars were trainees in Family Medicine.

## Research methods and design

### Study area and period

We conducted a study among medical practitioners at Odi District Hospital, Gauteng province, between 01 October and 31 October 2013.

### Study design

This was a cross-sectional study.

### Study population

At the time of the study, there were 50 medical practitioners at the hospital. They were all eligible for the study.

### Sampling procedure

Of the 50 medical practitioners eligible for the study, only 48 medical practitioners completed the questionnaire (response rate: 96%). The inclusion criteria were employment at Odi District Hospital, on a full-time or sessional basis. The period of the doctor’s employment at the hospital to qualify for inclusion was set at a minimum of 6 months, which the researchers regarded as adequate exposure for the medical practitioner to field questions in the questionnaire about his/her experience at the hospital. Other categories of health care professionals - pharmacists, for example - were excluded from the study. We recruited the medical practitioners on individual basis. The principal researcher outlined the aim, objectives and the study processes to each medical practitioner.

There were four categories of medical practitioners in the hospital: community service doctors (3), MOs (21), registrars (22) and others (2) – the latter referring to those in administrative posts, for example, the clinical manager.

### Data collection

A self-administered questionnaire was used to assess their knowledge, attitudes and management skills about overweight and obesity. The questionnaire was derived from a validated questionnaire.^29^ Given that a questionnaire developed in a different time, country or cultural context may not be a valid measure in a different population, questions that were not applicable in our setting, for example, ‘Pharmacotherapy for weight-management strategies reported by physicians’^30^ were removed since the medical practitioners in our setting did not prescribe medication for weight management. We piloted the questionnaire on five medical practitioners at Jubilee District Hospital located 50 km from the study site to establish the degree to which the questions elicited the same response among the respondents. Furthermore, at the point of questionnaire administration, the researcher requested each respondent to complete all the questions in that sitting rather than having another completion opportunity in future settings, to minimise data entry errors.^30,31^ Data collected in the pilot study were excluded from the main study. However, the data collected through our questionnaire was not compared with data collected through similar questionnaires in other studies.

Regarding their knowledge on overweight and obesity, they were asked to indicate if they agreed, disagreed or were unsure about statements relating to overweight and obesity as a national problem in South Africa, medical practitioners’ role in overweight and obesity prevention, overweight and obesity definition in terms of the BMI, the goal in the management of overweight and obesity and the status of overweight and obesity management at Odi District Hospital in particular.

Regarding their attitudes towards management of patients with overweight or obesity, they were assessed on the basis of being a responsible professional in the management of overweight or obesity, the adequacy of the training on overweight and obesity management at undergraduate level, the level of health care at which overweight and obesity should primarily be managed, their confidence in the management of overweight and obesity in adult and paediatric patients, as well as their attitudes towards the success of overweight and obesity management.

Regarding their management skills with regard to overweight and obese patients, they were asked to indicate the extent to which they measured the BMI of patients with chronic diseases and overweight and obesity, provided lifestyle modifications and involved the patient’s family in the management of the patient’s overweight and obesity. Lifestyle modifications include low-calorie, low-fat diet and 150 minutes/week of physical activity.^32^ At the time of the study, BMI measurement was the responsibility of the medical practitioner.

### Data analysis

Baseline characteristics were presented as descriptive data in absolute numbers and frequencies. A comparison through bivariate data analysis was made between those who had received training in the management of overweight and obesity (per medical practitioners’ category) and those who had not. Training in overweight/obesity referred to ever attending a short course or a workshop on the management of overweight and obesity, including during undergraduate medical training. A similar data analysis process was conducted to determine the level of the medical practitioners’ knowledge, attitudes and management skills in the management of overweight/obesity. Finally, a comparison was made between medical practitioners who were pursuing a post-graduate training in Family Medicine (registrars) versus those who were not (non-registrars), in relation to the management of overweight and obesity among patients. The SPSS^®^ statistical software (Version 22) was used for data analysis. A *p* < 0.05 was considered significant.

## Ethical considerations

The Medunsa Research and Ethics Committee (MREC) approved the study and allocated the clearance certificate number MREC/M/27/2013:PG. The clinical manager at Odi Hospital gave permission to conduct the study at the site. All respondents provided written informed consent for the study. Participation was voluntary and the respondents were informed of their right to withdraw from the study at any stage without negative consequences. Anonymity and confidentiality of the collected data were also confirmed.

## Results

In Table 1, the baseline characteristics of the participants are documented and they show that the majority of the medical practitioners (21; 43%) were aged between 31 and 40 years. There were more male medical practitioners (28; 58.3%). Most of them (34; 70.8%) had graduated between the year 2001 and 2011. The MOs and registrars constituted the highest percentage (89.6%).

**TABLE 1:** Baseline characteristics (*n* = 48).

Baseline characteristics	Frequency	Percentage
Age (Years)		
21–30	7	14.6
31–40	21	43.7
41–50	12	25.0
51–60	7	14.6
Not indicated	1	2.1
Mean	40.3	-
Range	29–60	-
Standard deviation	8.6	-
Gender		
Female	18	37.5
Male	28	58.3
Not indicated	2	4.2
Graduation year grouped		
1982–1990	4	8.3
1991–2000	9	18.8
2001–2011	34	70.8
Not indicated	1	2.1
Professional categories		
Community service doctor	3	6.2
Medical officer	21	43.8
Registrar	22	45.8
Other	2	4.2

*Source*: Odi District Hospital records (2013) and medical practitioners

Table 2 shows that among the three community service doctors in the hospital, two (66.7%) had received training on the management of overweight and obesity (after qualifying as medical practitioners). The majority of the MOs (18; 85.7%) and registrars (17; 77.3%) responded that they had not received training in the management of overweight and obesity. In both cases, there was a significant difference between those who responded that they had received training versus those who reported to the contrary (*p* < 0.001). When all the medical practitioner categories were pooled together, there was a statistically significant difference between those who indicated that they had received training on overweight and obesity management (11; 22.9%) versus those who indicated that they had not (37; 77.1%), (*p* < 0.001).

**TABLE 2:** Proportion of medical practitioners by professional category on overweight/obesity management training.

Professional category (*n* = 48)	Received training on overweight/obesity management			
	Yes	No	Total	p*
Community service doctor	2 (66.7)	1 (33.3)	3 (100.0)	0.412
Medical officer	3 (14.3)	18 (85.7)	21 (100.0)	< 0.001
Registrar	5 (22.7)	17 (77.3)	22 (100.0)	< 0.001
Other	1 (50.0)	1 (50.0)	2 (100.0)	0.529
**Total**	**11 (22.9)**	**37 (77.1)**	**48 (100.0)**	**< 0.001**

*Source*: Odi District Hospital records (2013) and medical practitioners

* Fisher’s exact test.

Table 3 shows that 47 (97.9%) medical practitioners agreed with the statement that overweight and obesity was a problem in South Africa. Medical practitioners demonstrated poor knowledge on the classification of BMI (*p* < 0.05). Ten (20.8%) medical practitioners were unsure whether a sustained 10% body weight loss over 6 months was an important goal in the management of overweight and obesity.^33^ Forty (83.3%) medical practitioners disagreed that overweight and obesity management was effective at Odi Hospital.

**TABLE 3:** Medical practitioners’ knowledge on overweight/obesity.

Statements on knowledge assessment	Medical practitioners’ knowledge on overweight/obesity			
	Agree	Disagree	Unsure	Total	p*
Overweight/obesity is a problem in South Africa. *n* (%)	47 (97.9)	1 (2.1)	0 (0.0)	48 (100.0)	< 0.001
The role of medical practitioners in the prevention of overweight/obesity is limited. *n* (%)	20 (41.7)	26 (54.2)	2 (4.1 )	48 (100.0)	0.219
Most people can reduce their weight and maintain it. *n* (%)	24 (50.0)	20 (41.7)	4 (8.3)	48 (100.0)	0.412
Obesity is a BMI > 25 kg/m^2^. *n* (%)	29 (61.7)	17 (36.2)	2 (4.1)	48 (100.0)	0.014
Class I obesity is a BMI = 25–29 kg/m^2^.	29 (61.7)	15 (31.9)	4 (8.3)	48 (100.0)	0.004
Class II obesity is a BMI = 30–34.9 kg/m^2^. *n* (%)	32 (66.7)	12 (25.0)	4 (8.3)	48 (100.0)	< 0.001
A sustained 10% body weight loss is an important goal in the management of overweight/obesity. *n* (%)	32 (66.7)	6 (12.5)	10 (20.8 )	48 (100.0)	< 0.001
Anti-overweight/obesity medication should be started from a BMI of 25 kg/m^2^. *n* (%)	5 (10.4)	38 (79.2)	5 (10.4)	48 (100.0)	< 0.001
Overweight/obesity management is effective at Odi Hospital. *n* (%)	1 (2.1)	40(83.3)	7 (14.6)	48 (100.0)	< 0.001

*Source*: Medical practitioners at Odi District Hospital

BMI = body mass index;

* Fisher’s exact test.

Table 4, which reflects medical practitioners’ attitudes, demonstrates that 32 (66.7%) medical practitioners disagreed that management of overweight and obesity was the responsibility of a dietician versus 10 (20.8%) who agreed, with a statistically significant difference between the two groups (*p* < 0.001). Forty-five (94.0%) medical practitioners disagreed with the statement that overweight and obesity management is the sole responsibility of a specialist in internal medicine. Thirty-seven (77.1%) medical practitioners disagreed that the undergraduate training on overweight and obesity management was adequate for them versus two (4.1%) who agreed (*p* < 0.001). Twenty-two (45.8%) medical practitioners agreed with the statement that overweight and obesity should be managed at the primary health care level versus 17 (35.4%) who disagreed (*p* = 0.298).

**TABLE 4:** Medical practitioners’ attitudes towards management of overweight/obesity (*n* = 48).

Statements on attitudes assessment	Medical practitioners’ attitudes towards management of overweight/obesity			
	Agree	Disagree	Neutral	Total	p*
Overweight/obesity management is mainly the responsibility of a dietician.	10 (20.8)	32 (66.7)	6 (12.5)	48 (100.0)	< 0.001
Overweight/obesity management is the sole responsibility of a specialist in internal medicine.	0 (0)	45 (94.0)	3 (6.3)	48 (100.0)	< 0.001
Overweight/obesity management was adequately covered during undergraduate medical training.	2 (4.1)	37 (77.1)	9 (18.8 )	48 (100.0)	< 0.001
Overweight/obesity should be managed at the primary health care level.	22 (45.8)	17 (35.4)	9 (18.8)	48 (100.0)	0.298
I am confident of managing overweight/obesity in adult patients.	21 (43.7)	26 (54.2)	1 (2.1)	48 (100.0)	0.308
I am confident of managing overweight/obesity in children.	12 (25.0)	35 (72.9)	1(2.1))	48 (100.0)	< 0.001
Management of obese patients is difficult for medical practitioners.	22 (45.8)	17 (35.4)	9 (18.8)	48 (100.0)	0.298
Overweight/obesity management is usually unsuccessful.	27 (56.2)	13 (27.1)	8 (16.7)	48 (100.0)	0.004
Medical practitioners lack interest in the management of overweight/obesity.	27 (56.2)	13 (27.1)	8 (16.7)	48 (100.0)	0.004

*Source*: Medical practitioners at Odi District Hospital

* Fisher’s exact test.

Medical practitioners did not differ significantly (*p* = 0.308) between those who were confident on the management of overweight and obesity among adult patients (21; 43.7%) versus those who were not (26; 54.2%). They differed significantly with regard to management of overweight and obesity among children (*p* < 0.001). There were more medical practitioners who agreed (22; 45.8%) that management of overweight and obesity was difficult for medical practitioners versus those who disagreed (17; 35.4%), but there was no statistically significant difference between the two groups. Twenty-seven (56.2%) medical practitioners agreed with the statement that overweight and obesity management was usually unsuccessful, while 13 (27.1%) disagreed (*p* = 0.004). The same results were obtained for the statement that medical practitioners lacked interest in the management of overweight and obesity.

The recording of patients’ weight as part of the management of patients with chronic diseases was done most of the time by 15 (31.3%) medical practitioners, while 28 (58.3%) indicated that they did so occasionally (*p* = 0.008). Twenty-nine (60.4%) medical practitioners indicated that they recorded patients’ weight as part of opportunity screening for overweight and obese patients versus 11 (22.9%) who did so occasionally (*p* < 0.001). Twenty-four (50%) medical practitioners indicated that they measured the weight of overweight and obese patients routinely versus 17 (35%) who did so occasionally – but there was no statistical difference between the two groups (*p* = 0.150).

Provision of lifestyle modifications as part of the management of patients with overweight and obesity was done most of the time by 47 (97.9%) medical practitioners versus 1 (2.1%) who did so occasionally (*p* < 0.001). Involvement of the patient’s family members in the management of the patient’s overweight and obesity was done most of the time by 43 (89.6%) medical practitioners, while 3 (6.3%) did so occasionally (*p* < 0.001) (Table 5).

**TABLE 5:** Medical practitioners’ management skills for patients with overweight/obesity (*n* = 48).

Statements on management skills assessment	Medical practitioners’ skills for patients with overweight/obesity			
	Most of the time	Occasionally	Never	Total	p*
I measure the BMI as part of management of patients with chronic disease. *n* (%)	15 (31.3)	28 ( 58.3)	5 (10.4)	48 (100.0)	0.008
I measure the BMI as part of opportunistic screening on overweight/obese patients. *n* (%)	11 (22.9)	29 (60.4)	8 (16.7)	48 (100.0)	< 0.001
I measure the BMI of overweight/obese patients as routine. *n* (%)	24 (50.0)	17 (35.4)	7 (14.6)	48 (100.0)	0.150
I provide lifestyle modifications as part of management of patients with overweight/obesity. *n* (%)	47 (97.9)	1 (2.1)	0 (0)	48 (100.0)	< 0.001
I involve family members in the management of patients with overweight/obesity. *n* (%)	43 (89.6)	3 (6.3)	2 (4.1)	48 (100.0)	< 0.001

*Source*: Medical practitioners at Odi District Hospital

* Fisher’s exact test.

Table 6 shows that there were significantly more non-registrars 11 (52.4%) versus registrars 4 (20.0%) who indicated that they recorded weight as part of management of patients with chronic diseases (*p* = 0.033). There were also significantly more non-registrars 14 (77.8%) versus registrars 8 (38.1%) who measure weight for overweight patients routinely (*p* = 0.013). There were no other significant differences in knowledge, attitudes and practice relating to the management of overweight and obesity between registrars and non-registrars (*p* > 0.050).

**TABLE 6:** Comparison of knowledge, attitudes and skills regarding overweight/obesity management between registrars and non-registrars.

Statement	Response	Registrars *n*(%)	Non-registrars *n*(%)	p*
Knowledge				
Overweight/obesity is a problem in South Africa ( *n*= 44).	Yes No	22 (100) 0(0.0)	23 (95.8) 1(4.2)	0.338 -
Obesity is a BMI > 25 kg/m^2^ ( *n*= 44).	Yes No	11 (50.0) 11(50.0)	16 (72.7) 6(27.3)	0.126 -
Class I obesity is a BMI = 25–29.9 kg/m^2^ ( *n*= 43).	Yes No	11 (52.4) 10(47.6)	17 (77.3) 5(22.7)	0.090 -
A sustained 10% body weight loss over 6 months is an important goal in the management of overweight/obesity ( *n*= 36).	Yes No	13 (81.3) 3(18.7)	18 (90.0) 2(10.0)	0.457 -
Attitudes				
Medical practitioners have a limited role in the management of overweight/obesity ( *n*= 44).	Yes No	8 (38.1) 13(61.9)	12 (52.2) 11(47.8)	0.354 -
Management of overweight/obesity is the responsibility of a dietician ( *n*= 41).	Yes No	4 (22.2) 14(77.8)	5 (21.7) 18(78.3)	0.971 -
I am confident of managing overweight/obesity in adult patients ( *n*= 46).	Yes No	11 (52.4) 10(47.6)	10 (41.7) 14(58.3)	0.477 -
I am confident of managing overweight/obesity in children ( *n*= 45).	Yes No	5 (23.8) 16(76.2)	3 (15.0) 17(85.0)	0.482 -
Management skills				
I measure the BMI as part of management of patients with chronic disease ( *n*= 41).	Yes No	4 (20) 16(80)	11 (52.4) 10(47.6)	0.033 -
I measure the BMI of overweight patients as routine ( *n*= 39).	Yes No	8 (38.1) 13(61.9)	14 (77.8) 4(22.2)	0.013 -
I measure the BMI of obese patients as routine ( *n*= 38).	Yes No	11 (47.6) 10(47.6)	11 (64.7) 6(35.3)	0.450 -
The weight management programme always includes referring to a dietician ( *n*= 46).	Yes No	15 (68.2) 7(31.8)	20 (87.0) 3(13.0)	0.134 -
I always include advice on physical activity in weight management ( *n*= 44).	Yes No	22 (100.0) 0(0)	24 (100.0) 0(0)	0.110 -

*Source*: Medical practitioners at Odi District Hospital

* Fisher’s exact test.

## Discussion

This study has shown that there was a significantly fewer number of medical practitioners who had received training in weight management. A high percentage (97.9%) of the medical practitioners were aware that overweight and obesity are a problem in South Africa, but >60% of them did not know the classification of overweight and obesity according to the BMI. Their attitudes were that weight management was not primarily the responsibility of a dietician or a specialist in internal medicine. However, they were not confident in the management of overweight and obesity in children. They regarded weight management as usually unsuccessful and regarded themselves as lacking interest in weight management. They occasionally (versus ‘most of the time’) practised BMI measurement but provided lifestyle modifications and involved the patient’s family in weight management most of the time. More non-registrars compared with registrars measured patients’ BMI as part of the management of patients with chronic diseases and as routine for overweight patients.

In our study, almost all the medical practitioners (97.9%) were aware that overweight and obesity was a problem in South Africa. Indeed, a study has shown that in South Africa, the prevalence of overweight and obesity increased significantly from 23.5% in 2008 to 27.2% in 2012 (*p* < 0.001).^34^ More than 60% of the medical practitioners demonstrated good knowledge on the classification of BMI. This was higher than the percentage obtained by Block et al. in their survey of residents in internal medicine that revealed that only 40% displayed good knowledge about BMI.^35^ In a study conducted in Hungary on the evaluation of the knowledge, practice and attitudes of medical practitioners, it was found that awareness of the diagnostic threshold for overweight and obesity in terms of the BMI was 51% among general practitioners (an equivalent of MOs in our study) and 90% among residents (an equivalent of registrars in our study).

In this study, two-thirds of the medical practitioners indicated that overweight and obesity management was not primarily the responsibility of a dietician, and about nine-in-ten disagreed that overweight/obesity management was solely the responsibility of a specialist in internal medicine. Indeed, the management of overweight and obesity has been found to have a wider objective than weight loss alone – it includes mortality risk reduction and improvement of the quality of life.^36^ Therefore, appropriate management of overweight and obesity should be interdisciplinary, including lipid profile control, blood pressure management, evaluation of respiratory disorders (e.g. sleep apnoea syndrome), pain control for arthritis and management of psychosocial disturbances (e.g. eating disorders).^35^

More than three-quarters of the medical practitioners disagreed that overweight and obesity management were adequately covered at undergraduate medical training. In its report referred to earlier in this article, The Royal College of Physicians has indicated that the minimal and poorly coordinated training of medical practitioners on weight management reflected lack of training on overweight and obesity at undergraduate level.^23^

More medical practitioners (45.8%) expressed difficulty in managing overweight and obesity. This has been shown to be related mainly to three barriers in the initiation of the topic: lack of competence on overweight and obesity care, concern about possible negative consequences elicited by introducing the topic to patients during a consultation and time constraints and limited resources to raise the sensitive topic,^37^ especially if the overweight and obesity are not the presenting complaint in a given consultation. Since Odi District Hospital is a primary health care facility with a large workload of patients, the authors are of the opinion that the medical practitioners had a similar explanation for the difficulty they expressed in the management of overweight and obesity.

Schmidt et al. in their study on how weight management specialists treated overweight and obesity before 2012 discovered that 99% of them used stimulant medication (amphetamines, diethylpropion and phendimetrazine) without time limit on amphetamines (50%) and at higher doses than recommended in the package insert.^38^ In our study, we did not require the medical practitioners to provide us with the medication names. According to the knowledge of the authors, weight control medication is excluded from the medical formulary of the South African public sector health care facilities.^39^ Therefore, at Odi District Hospital, each practitioner would refer patients in need mainly to the private sector facilities. The main emphasis on weight management in the public sector would be lifestyle modifications.^28^

More than half of the medical practitioners (54.2%) indicated that they were not confident on the management of overweight and obesity among adult patients, in keeping with the study by Block et al. where medical practitioners expressed negative opinions about their skills for treating obese patients.^35^ The percentage was significantly higher (72.9%) among medical practitioners who expressed lack of confidence in the management of childhood overweight and obesity. Several studies have indicated that overweight and obesity treatment could improve in primary care by supporting practitioners to identify and manage paediatric patients.^40,41,42^

In this study, about half (56.2%) of the medical practitioners viewed management of overweight and obesity as usually unsuccessful, in keeping with a study by Ferrante et al. in their survey of practices and attitudes of family physicians in New Jersey (USA), where 51% of the family physicians regarded treatment for overweight and obesity as often ineffective.^43^ Nevertheless, health care providers need to show interest in weight management as it has been demonstrated that patient behavioural change increases with provider encouragement and goal setting.^44^

Significantly, more medical practitioners (58%) recorded patients’ BMI occasionally (versus 31% who indicated ‘most of the time’ as part of the management of patients with chronic diseases. Overweight and obesity should be included among the list of chronic diseases as it is a component of the metabolic syndrome, the other components being an increased WC, elevated triglycerides, reduced high-density lipoprotein (HDL) cholesterol, elevated blood pressure and elevated fasting glucose.^45^ Successful overweight and obesity management, as chronic diseases, has been shown to improve the quality of life. This has been demonstrated in the study by Sarwer et al. conducted at the University of Pennsylvania (United States) where a mean weight loss of 3.9 ± 0.3 kg was associated with improvement in the quality of life.^45^

Our study demonstrated that almost 98% of medical practitioners indicated that they provided lifestyle modifications as part of the management of patients with overweight and obesity. The effect of this intervention, albeit only verbal, should not be underestimated as it has been shown that advice on losing weight by a health care professional was associated with the desire and attempt to lose weight (OR = 3.71 and OR = 3.53, 95% CI, respectively) among overweight and obese British adults – independent of demographic and weight status.^46^

Almost 90% of medical practitioners indicated that they involved the patient’s family members most of the time in the management of the patient’s overweight and obesity. It has been shown that social support from family and friends is effective in assisting patients to shed weight and prevent weight gain.^47,48^

There were significantly more non-registrars (52.4%) versus registrars (20.0%) who indicated that they measured patients BMI as part of management of patients with chronic diseases, and also significantly more non-registrars (77,8%) who measured the BMI among overweight patients routinely. Weight loss in overweight and obese patients has been associated with improvement of chronic conditions such as asthma,^49^ CVD (including heart failure),^50^ and diabetes mellitus.^51^

Regarding BMI measurement, we would have expected an opposite finding in which registrars would have performed this measurement more than non-registrars, given that the former were undergoing post-graduate training in patient care. The explanation for this opposite finding is unclear to the authors. However, we are aware of the studies by Ferrante et al. in which they reported that family physicians expressed difficulty in conducting a physical examination (including weight management) in obese patients,^43^ and Rurik et al. who reported inconsistency among family physicians in Hungary in measuring patients’ anthropomorphic indices.^52^

### Recommendations

Given that overweight and obesity have become a global problem, the undergraduate medical curriculum planners should incorporate training in weight management in the undergraduate curriculum. Overweight and obesity should be treated as a non-communicable disease at undergraduate and post-graduate levels and in the medical practitioners’ fields of practice. Weight management should be a collaborative interdisciplinary team effort. Given the high percentage of medical practitioners who indicated lack of training in weight management, it is recommended that short courses and continuing medical education workshops be arranged on a mandatory basis for all medical practitioners. Such in-service training opportunities may address the lack in knowledge, correct attitudes and empower the medical practitioners to correctly apply the knowledge thus obtained.

### Strengths and limitations

To our knowledge, the study is the first to explore the knowledge, attitudes and management skills of medical practitioners regarding weight management in the whole of Tshwane District. However, the study was conducted in a single health care facility, which limits the generalisability of the study findings. The study was conducted among 48 (out of the available 50) medical practitioners who consented to participate at the study setting. Therefore, it is acknowledged that the sample size was relatively small. The data relating to the medical practitioners’ knowledge, attitudes and management skills were self-reported. Consequently, inaccuracies in the medical practitioners’ reporting cannot be excluded.

## Conclusion

The number of medical practitioners who had received training in weight management was significantly small. Almost all of the medical practitioners were aware of the problem of overweight and obesity in South Africa, but > 60% did not know the BMI classification of overweight and obesity. Their attitudes were that weight management was not mainly the responsibility of a dietician and not the sole responsibility of a specialist in internal medicine. Nevertheless, they were not confident in the management of overweight and obesity in children. They regarded weight management as usually unsuccessful and regarded themselves as lacking interest in weight management. They occasionally practiced BMI measurement but provided lifestyle modifications and involved the patient’s family in weight management most of the time. Non-registrars measured patients’ weight as part of the management of patients with chronic diseases and measured patients’ BMI routinely – significantly more than the registrars.

## References

[1] Rennie KL, Jebb SA Prevalence of overweight and obesity in Great Britain. Obes Rev. 2005;6:11–12. http://dx.doi.org/10.1111/j.1467-789X.2005.00164.x 15655034 10.1111/j.1467-789X.2005.00164.x

[2] Wang Y, Beydoun MA The obesity epidemic in the United States – Gender, age, socioeconomic, racial/ethnic, and geographic characteristics: A systematic review and meta-regression analysis. Epidemiol Rev. 2007;29:6–28. http://dx.doi.org/10.1093/epirev/mxm007 17510091 10.1093/epirev/mxm007

[3] Lim SS, Vos T, Flaxman AD, et al A comparative risk assessment of burden of disease and injury attributable to 67 risk factors and risk factor clusters in 21 regions, 1990–2010: A systematic analysis for the Global Burden of Disease Study 2010. Lancet. 2012;380:2224–2260. http://dx.doi.org/10.1016/S0140-6736(12)61766-8 23245609 10.1016/S0140-6736(12)61766-8 PMC4156511

[4] Olshansky SJ, Passaro DJ, Hershow RC, et al A potential decline in life expectancy in the United States in the 21st century. N Engl J Med 2005;352:1138–1145. http://dx.doi.org/10.1056/NEJMsr043743 10.1056/NEJMsr043743 15784668

[5] Dalal S, Beunza JJ, Volmink J, et al Non-communicable diseases in sub-Saharan Africa: What we know now. Int J Epidemiol. 2011;40:885–901. http://dx.doi.org/10.1093/ije/dyr050 21527446 10.1093/ije/dyr050

[6] Follow-up to the Political Declaration of the High-level Meeting of the General Assembly on the Prevention and Control of Non-Communicable Diseases [homepage on the internet] Geneva, Switzerland: World Health Assembly; 2013 [cited 2015 Dec 23]. Available from: http://apps.who.int/gb/ebwha/pdf_files/WHA66/A66_R10-en.pdf

[7] Mayosi BM, Flisher AJ, Lalloo UG, et al The burden of non-communicable diseases in South Africa. Lancet. 2009;374:934–947. http://dx.doi.org/10.1016/S0140-6736(09)61087-4 19709736 10.1016/S0140-6736(09)61087-4

[8] Ziraba A, Fotso J, Ochako R Overweight and obesity in urban Africa: A problem of the rich or the poor? BMC Public Health. 2009;9:465 http://dx.doi.org/10.1186/1471-2458-9-465 20003478 10.1186/1471-2458-9-465 PMC2803188

[9] Puoane T, Steyn K, Bradshaw D, et al Obesity in South Africa: The South African demographic and health survey. Obes Res Clin Pract. 2002;10:1038–1048. 10.1038/oby.2002.141 12376585

[10] Alaba OA, Chola L Prevalence of age-adjusted obesity in South Africa and the influence of social determinants: An ecological analysis. Lancet. 2013;381:S6 http://dx.doi.org/10.1016/S0140-6736(13)61260-X

[11] Garrow JS Obesity and related diseases. London: Churchill Livingstone, 1988; p. 1–16.

[12] WHO Obesity: Preventing and managing the global epidemic Report of a WHO Consultation (WHO Technical Report Series 894) [homepage on the internet]. [cited 2015 Dec 23] Available from: http://www.who.int/nutrition/publications/overweight/obesity/WHO_TRS_894/en/11234459

[13] Bacopoulou F, Efthymiou V, Landis G, et al Waist circumference, waist-to-hip ratio and waist-to-height ratio reference percentiles for abdominal obesity among Greek adolescents. BMC Pediatr. 2015;15:50 http://dx.doi.org/10.1186/s12887-015-0366-z 25935716 10.1186/s12887-015-0366-z PMC4446827

[14] Akintunde AA Epidemiology of conventional cardiovascular risk factors among hypertensive subjects with normal and impaired fasting glucose. S Afr Med J. 2010;100:594–597. http://dx.doi.org/10.7196/SAMJ.3180 20822649 10.7196/samj.3180

[15] Cornier MA, Despres JP, Davis N, et al Assessing adiposity: A scientific statement from the American Heart Association. Circulation. 2011;124:1996–2019. http://dx.doi.org/10.1161/CIR.0b013e318233bc6a 10.1161/CIR.0b013e318233bc6a 21947291

[16] InterAct C, Langenberg C, Sharp SJ, et al Long-term risk of incident type 2 diabetes and measures of overall and regional obesity: The EPIC-InterAct case-cohort study. PLoS Med. 2012;9:e1001230 http://dx.doi.org/10.1371/journal.pmed.1001230 22679397 10.1371/journal.pmed.1001230 PMC3367997

[17] Castillo C, Carnicero JA, de la Torre MA, et al Nonlinear relationship between waist to hip ratio, weight and strength in elders: Is gender the key? Biogerontology. 2015;16(5):685–692. http://dx.doi.org/10.1007/s10522-015-9582-z 10.1007/s10522-015-9582-z 25966877

[18] Dietz WH, Baur LA, Hall K, et al Management of obesity: Improvement of health-care training and systems for prevention and care. Lancet. 2015;385(9986):2521–2533. http://dx.doi.org/10.1016/S0140-6736(14)61748-7 25703112 10.1016/S0140-6736(14)61748-7

[19] Blane DN, Macdonald S, Morrison D, et al Interventions targeted as primary care practitioners to improve the identification and referral of patients with co-morbid obesity: A realist review protocol. Syst Rev. 2015;4:61 http://dx.doi.org/10.1186/s13643-015-0046-y 25927993 10.1186/s13643-015-0046-y PMC4426175

[20] Starfield B, Shi L, Macinko J Contribution of primary care to health systems and health. Milbank Q. 2005;83:457–502. http://dx.doi.org/10.1111/j.1468-0009.2005.00409.x 16202000 10.1111/j.1468-0009.2005.00409.x PMC2690145

[21] NHS Health Scotland: Health promoting health service [homepage on the internet] 2009 [cited 2015 Dec 23] Available from: http://www.healthscotland.com/documents/3274.aspx

[22] RCP Action on obesity: Comprehensive care for all [homepage on the internet]. London: Royal College of Physicians; 2013 [cited 2015 Dec 24]. Available from: https://www.rcplondon.ac.uk/projects/outputs/action-obesity-comprehensive-care-all

[23] Tomiyama AJ, Finch LE, Belsky ACI, et al Weight bias in 2001 versus 2013: Contradictory attitudes among obesity researchers and health professionals. Obesity. 2015;23(1):46–53. 25294247 10.1002/oby.20910

[24] Sodjinou R, Bosu W, Fanou N, et al Nutritional training in medical and other health professional schools in West Africa: The need to improve current approaches and enhance training effectiveness. Global Health Action. 2014;1–9. http://dx.doi.org/10.3402/gha.v8.29415 10.3402/gha.v7.24827 PMC4119290 25084833

[25] Duncan PR, Howe LD, Manukusa Z, et al Determinants of obesity and perception of weight in hypertensive patients in rural South Africa. S Afr J Clin Nutr. 2014;27(2):56–62. http://dx.doi.org/10.1080/16070658.2014.11734488

[26] Malan Z, Mash B, Everett-Murphy K Development of a training programme for primary care providers to counsel patients with risky lifestyle behaviours in South Africa. Afr J Prm Health Care Fam Med. 2015;7(1):Art. #819, 1–8. http://dx.doi.org/10.4102/phcfm.v7i1.819 10.4102/phcfm.v7i1.819 PMC4564846 26245608

[27] Malan Z, Mash B, Everett-Murphy K Evaluation of a training programme for primary care providers to offer brief behaviour change counselling on risk factors for non-communicable diseases in South Africa. Patient Educ Couns. 2016;99(1):125–131. http://dx.doi.org/10.1016/j.pec.2015.08.008 26324109 10.1016/j.pec.2015.08.008

[28] HPCSA Registrations 2013 [homepage on the internet]. [cited 2015 Dec 12]. Available from: http://www.hpcsa.co.za/Registrations/Criteria

[29] Al-Ghawi A, Uauy R Study of knowledge, attitudes and practices of physicians towards obesity management in primary health care in Bahrain. Public Health Nutr. 2009;12(10):1791–1798. http://dx.doi.org/10.1017/S1368980008004564 19161645 10.1017/S1368980008004564

[30] Cook TD, Campbell DT Quasi-experimentation: Design and analysis issues for field settings. Boston, MA: Houghton Mifflin Company; 1979.

[31] Pati S, Hussain MA, Swain S, et al Development and validation of a questionnaire to assess multimorbidity in primary care: An Indian experience. BioMed Res Int. 2016;2016: 1–9. 10.1155/2016/6582487 PMC4761379 26966687

[32] Wadden TA, Webb VL, Moran CH, et al Lifestyle modification for obesity. Circulation. 2012;125(9):1157–1170. http://dx.doi.org/10.1161/CIRCULATIONAHA.111.039453 22392863 10.1161/CIRCULATIONAHA.111.039453 PMC3313649

[33] NHLBI Obesity Education Initiative Expert Panel on the Identification, Evaluation, and Treatment of Obesity in Adults (US) Clinical guidelines on the identification, evaluation, and treatment of overweight and obesity in adults: The evidence report [homepage on the internet]. Bethesda, MD: National Heart, Lung, and Blood Institute; 1998 [cited 2016 June 27] Available from: http://www.ncbi.nlm.nih.gov/books/NBK2004/

[34] Sartorius B, Veerman LJ, Manyema M, et al Determinants of obesity and associated population attributability, South Africa: Empirical evidence from a national panel survey, 2008–2012. PLoS One. 2015;10(6):e0130218 http://dx.doi.org/10.1371/journal.pone.0130218 10.1371/journal.pone.0130218 PMC4463861 26061419

[35] Block JP, De Salvo KB, Fisher WP Are physicians equipped to address the obesity epidemic? Knowledge and attitudes of internal medicine residents. Prev Med. 2003;36(6):669–675. http://dx.doi.org/10.1016/S0091-7435(03)00055-0 12744909 10.1016/s0091-7435(03)00055-0

[36] Mariani S, Watanabe M, Lubrano C, et al Interdisciplinary approach to obesity [homepage on the internet]. [cited 2015 Dec 26] Available from: http://link.springer.com/chapter/10.1007/978-3-319-09045-0_28#page-1

[37] Blackburn M, Stathi A, Keogh E, et al Raising the topic of weight in general practice: Perspectives of GPs and primary care nurses. BMJ Open. 2015;5:e008546 http://dx.doi.org/10.1136/bmjopen-2015-008546 10.1136/bmjopen-2015-008546 PMC4538258 26254471

[38] Schmidt SL, Bryman D, Greenway FL, et al How physician obesity medicine specialists treated obesity before 2012 new drug approvals. Obes Surg. 2015;25:186–190. http://dx.doi.org/10.1007/s11695-014-1466-9 25344465 10.1007/s11695-014-1466-9

[39] Standard treatment guidelines and essential medicines list for South Africa Primary health care level, 2014 edition [homepage on the internet]. [cited 2016 Jul 26] Available from: http://www.up.ac.za/media/shared/62/ZP_Files/standard-treatment-guidelines.zp66671.pdf

[40] Owen SE, Sharp DJ, Shield JP The Bristol online obesity screening tool: Experience of using a screening tool for assessing obese children in primary care. Prim Health Care Res Dev. 2011;12:293–300. http://dx.doi.org/10.1017/S1463423611000132 22284945 10.1017/S1463423611000132

[41] Gance-Cleveland B, Gilbert LH, Kopanos T, et al Evaluation of technology to identify and assess overweight children and adolescents. J Spec Pediatr Nurs. 2010;15:72–83. http://dx.doi.org/10.1111/j.1744-6155.2009.00220.x 20074114 10.1111/j.1744-6155.2009.00220.x

[42] Smith AJ, Skow A, Bodurtha J, et al Health information technology in screening and treatment of child obesity: A systematic review. Pediatrics. 2013;131:e894–902. http://dx.doi.org/10.1542/peds.2012-2011 23382447 10.1542/peds.2012-2011

[43] Ferrante JM, Piasecki AK, Ohman-Strickland P, et al Family Physicians’ practices and attitudes regarding care of extremely obese patients. Obesity (Silver Spring). 2009;17(9):1710–1716. http://dx.doi.org/10.1038/oby.2009.62 19282824 10.1038/oby.2009.62 PMC2953252

[44] Nelson JM, Vos MB, Walsh SM, et al Weight management-related assessment and counseling by primary care providers in an area of high childhood obesity prevalence: Current practices and areas of opportunity. Child Obes. 2015;11(2):194–201. http://dx.doi.org/10.1089/chi.2014.0052 25585234 10.1089/chi.2014.0052 PMC4382824

[45] Sarwer DB, Moore RH, Lisa K, et al The impact of a primary care-based weight loss intervention on quality of life. Int J Obes (Lond). 2013;37(Suppl 1):S25–S30. 23921778 10.1038/ijo.2013.93 PMC3786773

[46] Jackson SE, Wardle J, Johnson F, et al The impact of a health professional recommendation on weight loss attempts in overweight and obese British adults: A cross-sectional analysis. BMJ. 2013;3:e003693 10.1136/bmjopen-2013-003693 PMC3822310 24189083

[47] Keller C, Ainsworth B, Records K, et al A comparison of a social support physical activity intervention in weight management among post-partum Latinas. BMC Public Health. 2014;19(14):971 http://dx.doi.org/10.1186/1471-2458-14-971 10.1186/1471-2458-14-971 PMC4177167 25233867

[48] Wang ML, Pbert L, Lemon SC Influence of family, friend and coworker social support and social undermining on weight gain prevention among adults. Obesity (Silver Spring). 2014;22(9):1973–1980. http://dx.doi.org/10.1002/oby.20814 24942930 10.1002/oby.20814 PMC4435839

[49] Ma J, Strub P, Xiao L, et al Behavioural weight loss and physical activity intervention in obese adults with asthma. A randomised trial. Ann Am Thorac Soc. 2015;12(1):1–11. http://dx.doi.org/10.1513/AnnalsATS.201406-271OC 25496399 10.1513/AnnalsATS.201406-271OC PMC4342805

[50] Lavie CJ, Alpert MA, Ventura HO Risks and benefits of weight loss in heart failure. Heart Failure Clin. 2015;11:125–131. http://dx.doi.org/10.1016/j.hfc.2014.08.013 10.1016/j.hfc.2014.08.013 25432481

[51] Jackson SL, Long Q, Rhee MK, et al Weight loss and incidence of diabetes with the Veterans Health Administration MOVE! Lifestyle change programme: An observational study. Lancet Diabetes Endocrinol. 2015;3(3):173–180. http://dx.doi.org/10.1016/S2213-8587(14)70267-0 25652129 10.1016/S2213-8587(14)70267-0 PMC4401476

[52] Rurik I, Torzsa P, Ilyés I, et al Primary care overweight/obesity management in Hungary: Evaluation of the knowledge, practice and attitudes of family physicians. BMC Fam Pract. 2013;14:156 http://dx.doi.org/10.1186/1471-2296-14-156 24138355 10.1186/1471-2296-14-156 PMC3853764

